# A Retrospective Analysis of Spontaneous Adverse Drug Reactions Reports Relating to Paediatric Patients

**DOI:** 10.1371/journal.pone.0155385

**Published:** 2016-06-01

**Authors:** Rosliana Rosli, Long Chiau Ming, Noorizan Abd Aziz, Mohamed Mansor Manan

**Affiliations:** 1 Department of Pharmacy Practice, Faculty of Pharmacy, Universiti Teknologi MARA, Puncak Alam, Selangor, Malaysia; 2 Unit for Medication Outcomes Research and Education (UMORE), Pharmacy, School of Medicine, University of Tasmania, Hobart, Australia; 3 School of Pharmacy, KPJ Healthcare University College, Nilai, Negeri Sembilan, Malaysia; National Chiao Tung University, TAIWAN

## Abstract

**Background:**

Spontaneous reporting on adverse drug reactions (ADR) has been established in Malaysia since 1987, and although these reports are monitored by the Malaysia drug monitoring authority, the National Pharmaceutical Control Bureau, information about ADRs in the paediatric patient population still remains unexplored. The aims of this study, therefore, were to characterize the ADRs reported in respect to the Malaysian paediatric population and to relate the data to specific paediatric age groups.

**Methods:**

Data on all ADRs reported to the National Pharmaceutical Control Bureau between 2000 and 2013 for individuals aged from birth to 17 years old were analysed with respect to age and gender, type of reporter, suspected medicines (using the Anatomical Therapeutic Chemical classification), category of ADR (according to system organ class) as well as the severity of the ADR.

**Results:**

In total, 11,523 ADR reports corresponding to 22,237 ADRs were analysed, with half of these reporting one ADR per report. Vaccines comprised 55.7% of the 11,523 ADR reports with the remaining being drug related ADRs. Overall, 63.9% of ADRs were reported for paediatric patients between 12 and 17 years of age, with the majority of ADRs reported in females (70.7%). The most common ADRs reported were from the following system organ classes: application site disorders (32.2%), skin and appendages disorders (20.6%), body as a whole general disorders (12.8%) and central and peripheral nervous system disorders (11.2%). Meanwhile, ADRs in respect to anti-infectives for systemic use (2194/5106; 43.0%) were the most frequently reported across all age groups, followed by drugs from the nervous system (1095/5106; 21.4%). Only 0.28% of the ADR cases were reported as fatal. A large proportion of the reports were received from healthcare providers in government health facilities.

**Discussion:**

ADR reports concerning vaccines and anti-infectives were the most commonly reported in children, and are mainly seen in adolescents, with most of the ADRs manifesting in skin reactions. The majority of the ADR reports were received from nurses in the public sector, reporting ADRs associated with vaccine administration. The low fatality rate of ADR cases reported could potentially be caused by reporting bias due to the very low reporting percentage from the private healthcare institutions. This study indicates that ADR rates among Malaysian children are higher than in developed countries. Constant ADR reporting and monitoring, especially in respect to paediatric patients, should be undertaken to ensure their safety.

## Background

Adverse drug reaction (ADR) is defined as any response to a drug which is noxious and unintended, and which occurs at doses normally used in humans for prophylaxis, diagnosis, or therapy of disease, or for the modification of physiological function [1]. ADRs in children are not uncommon, with the literature showing that the incidence of ADRs in children is around 9.5%, with serious reactions accounting for 12% of the total number of ADRs [2]. Despite the fact that ADRs commonly occur in children (about three times more frequently than in non-elderly adults), there is little information regarding the characteristics of ADRs in this population [3].

The information on ADRs provided by the pharmaceutical companies during the premarketing phase of drug development is inevitably minimal. Since clinical trials are conducted in a limited timeframe, some ADRs, especially serious and latent ones, may not have taken place. As a consequence, only mild/moderate or non-serious ADRs tend to be captured during the development phase[4]. In addition, due to ethical and safety issues, vulnerable populations such as children, pregnant women and the elderly are rarely included in clinical trials [5–7], raising concerns about the safety profile of these drugs in these population groups [8].

Information on the safety profiles of drugs used in real healthcare settings is, therefore, very important in assisting healthcare professionals in making clinical decisions. This information can be acquired through the reporting of ADRs and it is, therefore, crucial to encourage healthcare professionals, and the public, to report any ADR events. In order to develop a systematic database on ADRs, the WHO has initiated an international ADR collaborative pharmacovigilance centre to monitor ADRs from all over the world. Systematic data collection from all collaborative centres enables the WHO to analyse adverse events associated with the use of drugs, identify signals or emerging problems and communicate how to minimise or prevent harm.

Pharmacovigilance is defined as “the science and activities relating to the detection, assessment, understanding and prevention of adverse effects or any other medicine-related problem” [9]. In Malaysia, pharmacovigilance activities are controlled by the National Centre for Adverse Drug Monitoring, a division within the National Pharmaceutical Control Bureau (NPCB) [10]. Unlike in other countries, ADR reporting in Malaysia is done voluntarily through spontaneous reporting by healthcare professionals, medical support staff, pharmaceutical companies and consumers. The submission of ADR reports can be done online or manually to the secretariat in the NPCB. Since the establishment of this process in 1987, a growing number of ADRs have been reported, largely due to increased awareness of the importance of ADR reporting [10].

During the study period, the total population of Malaysia comprised approximately 29.7 million inhabitants and it is estimated that 25% of these were aged from birth to 17 years old [11]. Since Malaysia is one of the countries within the WHO International Programme for International Drug Monitoring, the National Pharmacovigilance Centre should optimally send over 200 reports per million inhabitants per year to the WHO Collaborating Centre for International Drug Monitoring [12]. NPCB receives approximately 9000 to 11500 ADR reports annually, corresponding to between 300 and 387 reports per million inhabitants per year.

Although studies have been conducted on the ADRs in children reported to the international and national databases, the findings cannot be generalized to Malaysian children [13–18]. The possible reasons for these divergent findings are differences in settings, time periods, patient populations of different sizes and age groups, and different types of reporters [17]. In addition, the Malaysian healthcare system is different from that of many other countries.

Like other countries, the Malaysian healthcare system consists of two main sectors, namely the public and the private sectors. Nevertheless, the health system varies considerably from other countries in Southeast Asia since Malaysia has centralized the administration of its public sectors, which means that all the policy and programmes are centrally formulated and financed by the Ministry of Health. The public sector is fully funded by taxation and plays a major role in providing universal health services in Malaysia. In other words, patients who seek medical treatment from the government health facilities will only have to pay minimal fees as low as one Ringgit Malaysia (Malaysian currency which equivalent is to US$0.24), since other medical expenses, including the cost of the medications themselves are subsidized by the government. On the other hand the private sector is totally self-funded and patients need to pay for all their medical expenses, either by private health insurance or from their own pockets [19]. Patients who seek medical treatment in private health facilities have higher expectations and are more demanding since they are paying directly for their healthcare costs. Because of this, the private health facilities need to maintain their prestige in order to retain their current patients and attract new patients to their facilities.

To date, limited studies have been conducted on ADRs in Malaysian children. The aims of this study, therefore, were to characterize the ADRs reported in the Malaysian paediatric population and to relate the data to specific paediatric age groups. In this context, this study makes a crucial contribution to the provision of a detailed insight into the types of ADRs in Malaysian children by analysing ADRs in children reported to the Malaysian National Centre for Adverse Drug Reaction with respect to age and gender, type of reporters, suspected medicines, category of ADRs and severity of ADRs.

## Methods

### Setting

This was a retrospective study using the Malaysian ADR reports database, QUEST2. It is a repository governed by the National Centre for Adverse Drug Monitoring, National Pharmaceutical Control Bureau which is under the purview of Ministry of Health of Malaysia. The database or any data derived from QUEST2 are protected by relevant Malaysian laws (Control of Drugs and Cosmetics Regulation 1984). Researchers that are interested accessing this database could do so by obtaining permission from National Centre for Adverse Drug Monitoring. The QUEST2 system contains all spontaneous ADR reports in Malaysia, including those reported by healthcare professionals in government and/or private health facilities; other health care professionals, pharmaceutical companies and consumers. As of January 2014, the QUEST2 database contained almost 62,000 ADR reports received from all over Malaysia’s thirteen states and two federal territories. ADR reports submitted to the NPCB must include the following information: patient’s particulars, the suspected medicine(s), the presumed ADR(s) and the reporter’s details. Upon receipt of ADR reports, the information in the reports is assessed by trained staff at NPCB and all the findings in all reports are discussed at a meeting of the Malaysian Adverse Drug Reactions Advisory Committee prior to submission to the national drug control authority in Malaysia and to the World Health Organisation-Uppsala Monitoring Centre (WHO-UMC) situated in Sweden.

Approval to conduct this study was obtained from the National Institute of Health and Medical Research and Ethics Committee in the Ministry of Health, prior to implementation of the study (NMRR-14-1231-21610). Permission to access to the QUEST2 database was also granted. The data were retrieved from the QUEST2 system according to the ADR registration number that had been assigned automatically upon data entry into the database system. No informed consent were obtained prior to analysis as the patient information was already anonymized upon data entry to the database system.

### Data Extraction

All national pharmacovigilance centres (of which there are now more than 80 from all parts of the world) may either use WHO Adverse Drug Reactions Terminology (WHO-ART) or Medical Dictionary for Regulatory Activities (MedDRA) terms or codes for reporting to the WHO global individual case safety report database system, Vigibase [20]. Since, the National Centre for ADR Monitoring in Malaysia has been using WHO-ART for system organ class (SOC) classification in their reporting to WHO-UMC, the same classification for SOC (WHO-ART) was used in this study. Furthermore, as one of the member countries participating in the international drug monitoring programme, NPCB utilized the WHO-UMC causality assessment system for evaluation of ADR reports.

Data were extracted from the QUEST2 ADR database into Microsoft^®^ Excel files using the following criteria: Anatomical Therapeutic Chemical (ATC) classification of medications, active substance of the medicines, ADRs coded according to WHO Adverse Reaction Terminology (WHO-ART) at the SOC level, patient’s details (age and gender), type of reporters and severity of ADRs. Severity is defined as the intensity of a specific ADR event and can be categorized as mild, moderate, severe and fatal [21]. For the purpose of this study, in order to present the large amount of data in a comprehensive way, the medicines about which the reported ADRs were presented at ATC level 2.

The material comprised all ADR reports on children from birth to 17 years of age reported to the QUEST system from 2000 to 2013. These ADR reports were then subdivided according to the age categories based on the International Conference on Harmonisation Guidelines on Clinical Investigation of Medicinal Products in the Paediatric Population: neonates ≤ 27 days; infants ≤ 23 months; children 2–11 years; adolescents 12–17 years [22].

### Reports Excluded

Screening for possible duplicates in the ADR registration number was done using the duplication tool application in Microsoft Excel 2013. Duplication of reports can occur in large compilations of data when reports of the same events are being entered by more than one person. ADR reports relating to non-Malaysian citizens and those that had not been reviewed by the Malaysian Adverse Drug Reactions Advisory Committeewere excluded from the study. Other ADR reports that were excluded were reports on food supplements, traditional and veterinary products. Reports with no information on patient’s age were also excluded from this study, along with ADRs caused by drug administration to mothers. In order to enhance the quality of the data included in this study, only ADRs with causality assessed as certain, probable or likely and possible were analysed.

### Statistics

Data on ADRs according to years, gender, age group, type of reporters, suspected medicines and category of ADRs were described as frequencies and percentages. The analysis was undertaken using Microsoft Excel 2013 and IBM SPSS Version 19.

## Results

### Overall Characterization of QUEST2 Reports

**Fig 1:**
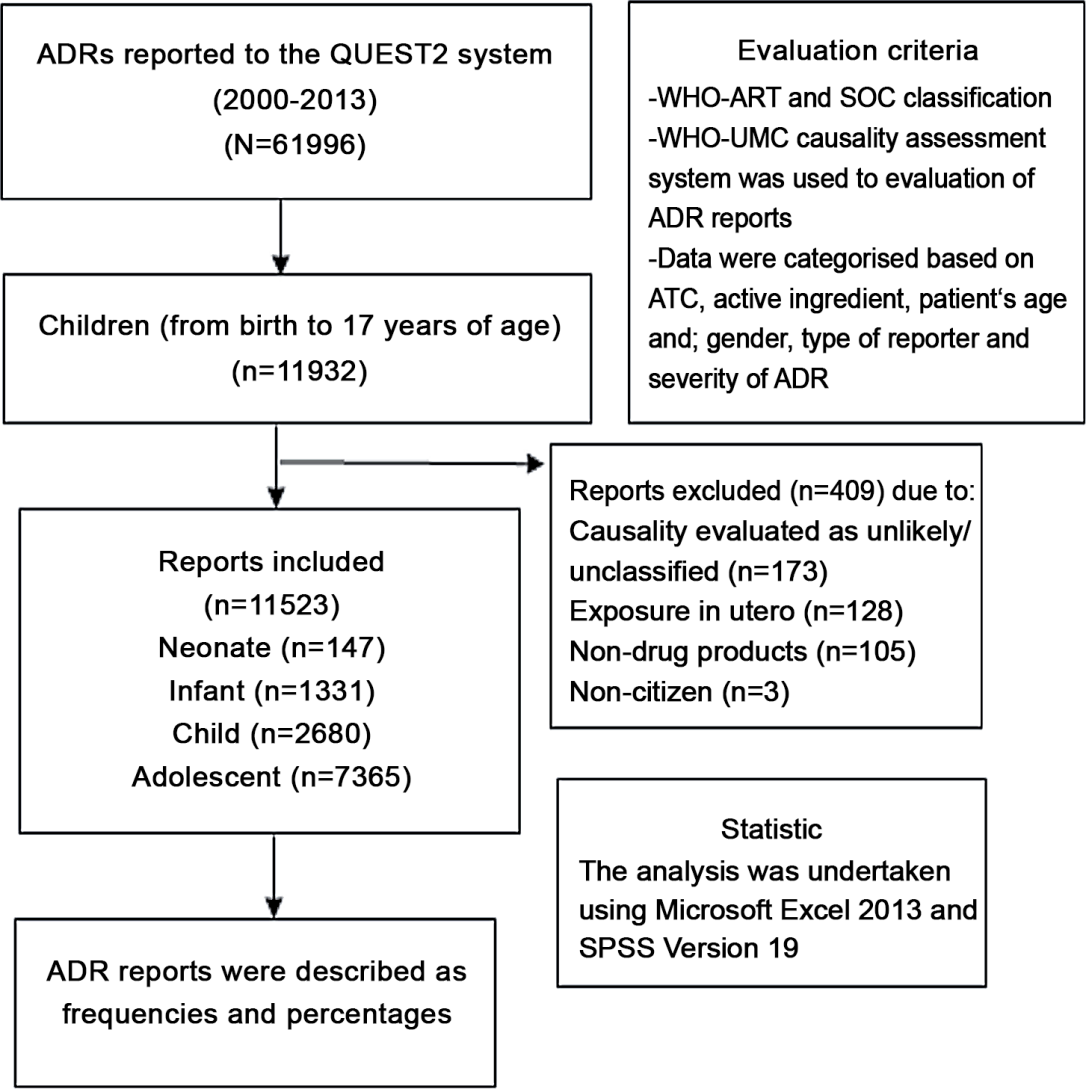
Flow chart of methodology and ADR reports included in the present study.

From 2000 to 2013, NPCB received a total of 61996 reports from various sources all over Malaysia. Of these, 19.2% (n = 11932) were related to children. The following reports: ADRs with causality evaluated as either unlikely, conditional or unclassified and unassessable or unclassifiable (n = 173), ADRs due to drug administration to the mother (n = 128), non-citizen (n = 3) and non-drug products (n = 105) were excluded from the study. After exclusion of these reports, a total of 11523 (18.6%) reports remained for use in this analysis (refer to Fig 1).

The 11523 reports received reported a total of 22237 ADRs for individuals from birth to 17 years of age during the study period. On average, from 2000 to 2007, 195 reports (range 97 to 305) were submitted per year, meaning that the overwhelming majority of reports have been submitted in recent years, with a peak in 2011 (n = 3527). The northern region of Malaysia (covering the states of Kedah, Perlis, Penang and Perak) reported the most ADRs (53.3%) followed by the central region (covering the state of Putrajaya, Kuala Lumpur, Selangor; 18.4%) and the East Coast Region (covering the states of Terengganu, Pahang, Kelantan; 10.4%). The highest number of ADRs reports was received from the state of Penang (42.4%).

Half of the reports received by the NPCB documented one ADR per report. Reports with ≥ 5 types of ADRs were commonly reported in adolescents in the 12–17 year age group. More than half (55.7%) of the ADRs reported in children were related to vaccines. In the reports where gender was known, 70.7% of the child reports concerned females. In general, female children (73.0%) were commonly seen to experience ADRs related to vaccines while the males (86.9%) were more prone to develop ADRs following drug administration.

### Gender and Age Group (Year 2000–2013 Comparison)

Table 1 illustrates a steadily increasing trend in the number of ADR reports from 2001 to 2010 and then a sharp increment in 2011 which levelled off thereafter. The majority of the reported ADRs occurred in adolescents aged between 12 to 17 years old (63.9%), whilst the fewest ADRs were reported in the neonatal group (1.3%). For vaccines, ADRs were most commonly reported for adolescents aged between 12 and 17 years (88.3%), and more than 90% of these ADRs were experienced by female children. On the other hand, for drugs, ADRs were most frequently reported in children aged 2 to 11 years old (45.6%) and adolescents 12 to 17 years old (33.3%), with male children having a higher tendency to develop ADRs (55.2%).

**Table 1:** Number of ADRs by year, gender and age groups.

Variables	0–27 days	28 days - 23 months	2–11 years	12–17 years	Combined
**ADR by year, n**					
**2000**	1	19	70	25	115
**2001**	1	22	52	22	97
**2002**	3	43	53	32	131
**2003**	6	50	65	40	161
**2004**	4	49	119	49	221
**2005**	11	66	134	94	305
**2006**	8	47	109	82	246
**2007**	3	52	138	93	286
**2008**	13	100	205	154	472
**2009**	19	135	287	194	635
**2010**	15	157	282	579	1033
**2011**	26	182	336	2983	3527
**2012**	0	100	383	1770	2253
**2013**	37	309	447	1248	2041
**ADR reports, n (%)**	147 (1.3)	1331 (11.5)	2680 (23.3)	7365 (63.9)	**11523 (100)**
**Gender, n (%)**					
**Vaccines, total**	15 (0.2)	387 (5.9)	352 (5.4)	5663 (88.3)	6417 (100)
Female	6 (0.1)	169 (2.8)	141 (2.4)	5633 (94.7)	5949 (92.7)
Male	8 (1.9)	205 (48.3)	190 (44.8)	21 (5.0)	424 (6.6)
Unknown	1 (2.3)	13 (29.5)	21 (47.7)	9 (20.5)	44 (0.7)
**Drugs, total**	132 (2.6)	944 (18.5)	2328 (45.6)	1702 (33.3)	5106 (100)
Female	55 (2.5)	385 (17.6)	954 (43.4)	802 (36.5)	2196 (43.0)
Male	71 (2.5)	541 (19.2)	1332 (47.3)	876 (31.1)	2820 (55.2)
Unknown	6 (6.7)	18 (20.0)	42 (46.7)	24 (26.7)	90 (1.8)

### Type of Reporter

More than 90% of the reports were sent by healthcare providers in government health facilities. Most of the reports were sent by nurses (36.7%) followed by pharmacists (30.2%) and doctors (21.8%). Other reporters consist of product holders (5.2%) and non-healthcare professionals (0.2%). There were only three reports from consumers. At the early phase of ADR reporting in Malaysia, most of the reports came from the doctors but the total number of reports from doctors has increased only marginally over the years. Reports received from pharmacists have increased significantly, however, until a peak in 2011. Reports from pharmacists dropped in 2012 before rising again in 2013. Reports from nurses, however, increased the most dramatically, with the first reports received from them in 2009, rising to a peak in 2011 before falling off.

### System Organ Class

From the 11523 reports received by the NPCB, 22237 SOCs were reported. “Application site disorders’ (32.2%) were the most commonly reported for children, followed by Skin and Appendages Disorders (20.6%), Body as a Whole General Disorders (12.8%), Central and Peripheral Nervous System Disorders (11.2%) and Gastrointestinal Disorders (8.1%). To make the findings more meaningful, the SOCs for Drugs and Vaccines were analysed separately according to children’s age groups.

Table 2 provides an overview of the reported ADRs related to vaccines and drugs classified by SOCs and distributed by age group. The total numbers of reported ADRs relating to vaccines and drugs are shown for all SOCs and age groups. From 6417 ADR reports received regarding vaccines, there were 13574 SOCs reported in the last 14 years. The most commonly reported SOCs for vaccines were Application Site Disorders (52.1%) and the other frequently reported reactions were injection site pain, injection site reaction, injection site rash and injection site pruritus Other common SOCs reported with their associated reactions were central and peripheral nervous system disorders (13.0%) (examples: dizziness and headache), body as a whole general disorders (12.5%) (examples: fever and asthenia), gastrointestinal system disorders (8.3%) (examples: nausea and vomiting) and musculo-skeletal system disorders (6.8%) (examples: myalgia and muscle weakness). All of these SOCs were mostly seen in adolescents aged 12 to 17 years old.

**Table 2:** System organ class for vaccines and drugs according to age group.

System Organ Class (SOC)	Age of Child				Total, n
	0-27days	28 Days—23 Months	2–11 Years	12–17 Years	
A) Vaccines					
Application Site Disorders	0	84	147	6845	7076
Central & Peripheral Nervous System Disorders	0	132	69	1568	1769
Body as a Whole General Disorders	7	195	230	1264	1696
Gastrointestinal System Disorders	2	56	40	1031	1129
Musculo-Skeletal System Disorders	0	7	4	910	921
Skin & Appendages Disorders	14	135	46	182	377
Psychiatric Disorders	0	16	10	346	372
Respiratory System Disorders	0	28	9	41	78
Secondary Term Events	0	5	36	1	42
Platelet, Bleeding & Clotting Disorders	0	8	8	5	21
White Cell & Res Disorders	0	17	1	2	20
Cardiovascular Disorders, General	0	11	3	4	18
Resistance Mechanism Disorders	0	11	5	1	17
Neonatal & Infancy Disorders	0	6	2	0	8
Heart Rate & Rhythm Disorders	0	1	0	5	6
Urinary System Disorders	0	1	4	1	6
Vision Disorders	0	0	0	4	4
Metabolic & Nutritional Disorders	0	3	0	1	4
Vascular (Extracardiac) Disorders	0	1	1	1	3
Endocrine Disorders	0	1	1	0	2
Red Blood Cell Disorders	0	1	0	1	2
Collagen Disorders	0	0	0	1	1
Hearing & Vestibular Disorders	0	1	0	0	1
Liver & Biliary System Disorders	1	0	0	0	1
**TOTAL**	**24**	**720**	**616**	**12214**	**13574**
B) Drugs					
Skin & Appendages Disorders	69	843	2016	1276	4204
Body as a Whole General Disorders	10	139	551	447	1147
Central & Peripheral Nervous System Disorders	10	41	321	356	728
Gastrointestinal System Disorders	10	88	302	277	677
Respiratory System Disorders	13	65	176	188	442
Urinary System Disorders	14	37	143	116	310
Psychiatric Disorders	3	16	112	85	216
Vision Disorders	0	11	88	65	164
Liver & Biliary System Disorders	0	17	38	64	119
Cardiovascular Disorders, General	10	29	46	23	108
Application Site Disorders	2	7	38	34	81
Heart Rate & Rhythm Disorders	5	18	23	31	77
Metabolic & Nutritional Disorders	1	5	39	23	68
Vascular (Extracardiac) Disorders	1	17	24	19	61
Platelet, Bleeding & Clotting Disorders	6	12	22	17	57
Musculo-Skeletal System Disorders	0	0	15	33	48
Red Blood Cell Disorders	1	2	13	15	31
White Cell & Res Disorders	1	2	10	18	31
Neonatal & Infancy Disorders	17	5	0	0	22
Resistance Mechanism Disorders	2	0	4	12	18
Reproductive Disorders, Female	0	1	2	10	13
Endocrine Disorders	1	1	2	6	10
Hearing & Vestibular Disorders	0	0	3	5	8
Secondary Term Events	1	0	5	2	8
Foetal Disorders	1	1	3	0	5
Special Senses Other Disorders	0	0	2	1	3
Reproductive Disorders, Male	0	0	2	1	3
Collagen Disorders	0	0	0	2	2
Myo-, Endo, Pericardial & Valve Disorders	0	0	1	0	1
Neoplasms	0	0	0	1	1
**TOTAL**	**178**	**1357**	**4001**	**3127**	**8663**

Up to the year 2013, there were 5106 reports received by the NPCB related to drugs, with 8663 SOCs reported, with these being most common in children aged between 2 and 11 years old (46.2%). Unlike vaccines, SOCs from skin and appendages disorders (48.5%) which frequently manifested as rash urticarial, rash maculo-papular, pruritus and rash erythematous were the most common SOCs experienced by Malaysian children. Other common SOCs and their associated reactions were body as a whole disorders (13.2%) (fever), central peripheral nervous system disorders (8.4%) (oculogyric crisis), gastrointestinal system disorders (7.8%) (vomiting and diarrhoea) and respiratory system disorders (5.1%) (dyspnoea).

### Anatomical Therapeutic Chemical Classification

The most frequently reported agents belonged to the ‘Anti-infective for systemic use’ category (74.7%), and almost half of the reports were associated with vaccines (55.7%). Of these, viral vaccines contributed 52.4% of the reports followed by bacterial and viral (combined) (2.0%) and bacterial vaccines (1.3%). The largest proportion of ADR reports were received for Human papillomavirus (HPV) vaccine(87.6%). ADRs on vaccines were most frequently reported by nurses (65.6%) followed by pharmacists (15.7%). In the next stage of analysis, reports on vaccines were excluded and reports on drugs were further analysed according to age group.

Table 3 displays the number of ADRs by therapeutic group (ATC Level 1) and age groups. Of the reported ADRs, 43% concerned Anti-infective for Systemic Use (ATC group J), which was the most frequently reported across all age groups, followed by drugs from the nervous system (ATC group N) (21.4%). Other therapeutic groups, namely Musculo-skeletal System (ATC group M) (7.6%), Alimentary Tract and Metabolism (ATC group A) (7.3%), Respiratory System (ATC group R) (7%) and Antineoplastic and Immunomodulating (ATC group L) (3.8%) were among the other common drugs reported for ADRs in Malaysian children.

**Table 3:** ADRs for drugs distributed by therapeutic group and age groups.

*Therapeutic Group (ATC Level 1)	Age of Child				Total, n
	0-27days	28 Days—23 Months	2–11 Years	12–17 Years	
Anti-infectives for Systemic Use	70	588	1010	526	2194
Nervous System	12	157	478	448	1095
Musculo-Skeletal System	3	20	147	216	386
Alimentary Tract & Metabolism	7	38	157	170	372
Respiratory System	16	58	209	75	358
Antineoplastic & Immunomodulating Agents	2	5	111	76	194
Various	0	7	58	50	115
Blood & Blood Forming Organs	2	8	34	34	78
Systemic Hormones Preparations (excluding Gender Hormones & Insulins)	0	11	32	33	76
Sensory Organs	10	34	18	8	70
Cardiovascular Systems	8	8	21	27	64
Dermatologicals	2	8	27	22	59
Antiparasitic Products, Insecticides & Repellents	0	3	25	11	39
Genito-Urinary System & Gender Hormones	0	0	1	5	6
**Total, n**	**132**	**945**	**2328**	**1701**	**5106**

*Note the exclusion vaccine reports in these counts

The data were further analysed according to the six most common therapeutic groups in children (ATC Level 2), as shown in Table 4. The most common therapeutic groups reported for ADRs were anti-bacterials for systemic use (39.2%) followed by analgesics (9.6%). Other common therapeutic groups are anti-inflammatory and anti-rheumatic products (6.8%), anti-epileptics (6.4%), anti-neoplastic agents (3.4%), drugs for functional gastrointestinal disorders (3.5%), psycholeptics (3.1%), drugs for obstructive airway disease (3.5%), antihistamines for systemic use (2.4%) and antivirals for systemic use (2%). In terms of age, children in the 2 to 11 years age group were most commonly reported to experience ADRs from all the frequently reported therapeutic groups except for ADRs associated with musculo-skeletal drugs (n = 216) and alimentary tract and metabolism drugs (n = 170) which were more dominant in adolescents in the 12–17 years age group.

**Table 4:** ADRs by common therapeutic groups (ATC Level 2) and age groups.

Therapeutic Group (ATC Level 2)	Age of Child				Total, n
	0-27days	28 Days—23 Months	2–11 Years	12–17 Years	
**Anti-infectives for Systemic Use**					
Antibacterials for systemic use	69	549	908	475	2001
Antivirals for systemic use	0	28	58	20	106
Immune sera & immunoglobulins	1	3	21	11	36
Antimycobacterials	0	2	12	14	28
Antimycotics for systemic use	0	6	11	6	23
**Total, n**	**70**	**588**	**1010**	**526**	**2194**
**Nervous System**					
Analgesics	3	118	223	146	490
Antiepileptics	2	26	146	155	329
Psycholeptics	4	9	48	99	160
Psychoanaleptics	2	2	41	31	76
Anesthetics	1	2	13	8	24
Other nervous system drugs	0	0	3	7	10
Antiparkinsons	0	0	3	2	5
**Total, n**	**12**	**157**	**478**	**448**	**1095**
**Alimentary Tract & Metabolism**					
Drugs for functional gastrointestinal disorders	2	14	83	79	178
Drugs for acid related disorders	3	3	21	41	68
Vitamins	1	13	23	12	49
Antidiarrheals, intestinal antiinflamatory/antiinfective agents	0	4	9	6	19
Mineral supplements	1	0	3	12	16
Drugs used in diabetes	0	2	4	10	16
Antiemetics & antinauseants	0	2	6	3	11
Drugs for constipation	0	0	4	4	8
Other alimentary tract & metabolism products	0	0	2	2	4
Stomatological preparations	0	0	1	1	2
Anabolic agents for systemic use	0	0	1	0	1
**Total, n**	**7**	**38**	**157**	**170**	**372**
**Musculo-skeletal System**					
Antiinflammatory & antirheumatic products	2	16	132	197	347
Muscle relaxants	1	4	8	10	23
Other drugs for disorders of the musculo-skeletal system	0	0	3	6	9
Drugs for treatment of bone disorders	0	0	3	1	4
Topical products for joint & muscular pain	0	0	1	2	3
**Total, n**	**3**	**20**	**147**	**216**	**386**
**Respiratory System**					
Drugs for obstructive airway diseases	4	36	111	27	178
Antihistamines for systemic use	6	16	71	31	124
Cough & cold preparations	1	5	20	15	41
Nasal preparations	0	1	7	1	9
Other respiratory system products	5	0	0	0	5
Throat preparations	0	0	0	1	1
**Total, n**	**16**	**58**	**209**	**75**	**358**
**Antineoplastic & Immunomodulating Agents**					
Antineoplastic agents	0	5	104	64	173
Immunosuppresants	1	0	6	6	13
Immunostimulants	1	0	1	6	8
Total, n	2	5	111	76	194

### Outcomes from ADRs

Over 14 years, only 6.8% (drug, n = 676; vaccines, n = 106) of reported ADRs were categorized as severe, with a large proportion of ADRs categorized as mild or moderate. In the reports where outcomes were known, the majority of the ADRs reported for drugs (69.2%) and vaccines (10.5%) the patients recovered without sequalae. At the time of reporting, there were quite a number of ADRs concerned with drugs (20.5%) and vaccines (3%) where the patient is not yet recorded as recovered. A small proportion of fatal cases of ADRs were reported for both drugs (n = 28) and vaccines (n = 4), as shown in Table 5.

**Table 5:** Characteristics of fatal ADRs.

*Suspected Agents (Drug/Vaccine)*	Age	Gender	Year	Adverse Reactions (WHO-ART)
**Caused by ADR**				
Ranitidine	6 years	Female	2000	Respiratory depression, cardiac arrest
Cisplatin	6 years	Male	2000	Steven Johnson Syndrome, death
Phenytoin	2 years	Female	2001	Bradycardia
Amoxycillin	4 years	Female	2007	Exanthema
Imatinib	17 years	Female	2005	Jaundice, sepsis
Suxamethonium	15 years	Male	2009	Hypothermia, tachycardia, acidosis metabolic, hyponatremia, hypertension
Azithromycin	15 years	Female	2008	Tachycardia ventricular
Pneumococcal vaccine	7 months	Male	2011	Infection streptococcal
**Drug Maybe Contributory**				
Beractant (4 cases)	1 day	3 female, 1 male	2011	Pulmonary haemorrhage
Metoclorpramide	1 month	Female	2011	Enterocolitis necrotising
Indomethacin	2 weeks	Female	2004	Renal function abnormal
Cyclophosphamide	1 year	Female	2012	Neutropenia
Promethazine	1 year	Male	2001	Face oedema, dyspnoea
Midazolam	7 years	Female	2003	Apnoea
Fentanyl	4 years	Male	2011	Bradycardia, hypotension
Doxorubicin	8 years	Female	2009	Cardiomyopathy
Diphenhydramine	2 years	Male	2008	Unconsciousness, seizure anoxic, respiratory depression, limpness body, muscle stiffness, cardiac arrest, eye rolling, breathing arrested
Carbamazepine immediate release tablet	14 years	Male	2009	Toxic epidermal necrolysis
Carbamazepine controlled release tablet	14 years	Male	2009	Toxic epidermal necrolysis
Sodium valproate	12 years	Male	2004	Renal failure, fever, jaundice, somnolence, rash erythematous, hepatic failure
Gentamicin (2 cases)	17 years	Male	2004	Renal failure acute
Lenograstim	17 years	Female	2006	Sepsis, Leukaemia acute megakaryocytic
Cloxacillin	14 years	Female	2007	Rash
Ceftriaxone	13 years	Male	2008	Hepatic enzymes increased
Meropenem	1 months	Male	2005	Diarrhoea, fever, colitis pseudomembranous, clostridial infection
DTP*-Hib + Hep B	3 months	Female	2008	Syncope, abnormal crying
DTP*-Hib + Polio	2 months	Male	2010	Abnormal crying hypotonic-hyporesponsive episode, fits, eyes gaze upward, fever
Hepatitis B	1 month	Female	2009	Vomiting, death

*DTP-Hib-Diphtheria, Tetanus, Pertussis and Haemophilus influenzae type b

Most fatal cases of ADRs were associated with the administration of Beractant (n = 4). Other commonly reported substances for fatal ADRs were Carbamazepine (n = 2), Bacterial and Viral Vaccines Combinations (n = 2) and Gentamicin (n = 2). Other substances, as depicted in Table 5, only reported a single fatal ADR case. Most fatalities were reported in adolescentsaged between 12 to 17 years old (n = 12). Children aged between 2 to 11 years and neonates reported the same number of fatalities while infants only reported four cases of fatalities.

## Discussion

### Overall Characterization of QUEST2 Reports

In order to facilitate standard practice across the country, public health facilities are obliged to adopt the policy and programmes set by the Ministry of Health. Apart from the limited policy and fiscal freedom afforded to the local managers, the public sector needs to achieve national performance indicators and targets that are linked to their annual budget. ADR reporting performance is one of the major national key performance indicators which is monitored by the higher authorities in the Ministry, in order to strengthen the pharmacovigilance activity in Malaysia. Unlike with the public sector, the Ministry of Health only has minimal regulatory power over the private sector [23]. Due to this lack of control over the private sector, the reporting of ADRs from private health facilities is lower than from government health facilities. In this study, more than 90% of the ADRs received were from the government health facilities. The sharp increment of ADR reporting observed from 2009 was mainly due to the surge in the number of pharmacists and nurses in public healthcare institutions [24]. Moreover, the types of drugs suspected to cause ADRs are different between the two systems since the private health facilities do not have to comply with the standard national drug formulary established by the Ministry of Health [23]. The different types of medications suspected to cause ADRs in these two forms of healthcare provision were not the focus of this study, however.

The ADR data for local children is very important and each country should have their own data on ADRs in this specific population group. Based on the national income level, classified in accordance with the World Bank Definition, Malaysia is an upper middle-income country [23]. High income countries, primarily in Europe and the US, have greater resources, competency and infrastructure to survey the safety of medicine. There is, therefore, far more information available about ADRs from high income countries. Conversely, there is only limited information about ADRs occurring in middle to low-income countries, including Malaysia [25]. Although many drugs have been extensively studied in the developed countries, their safety profile cannot necessarily be generalised to other countries, where the incidence, pattern and severity of adverse reactions may differ markedly because of local environmental and genetic influences [26, 27].

The data presented here shows that there are substantial numbers of suspected ADRs, 18.6% (vaccine: 10.4%, drugs: 8.2%) in children reported in Malaysia. This figure is close to the incidence of patients with ADRs reported in a study conducted at a public hospital located in the central region of Malaysia (16.5%) [28]. The proportion of ADRs in children from other international pharmacovigilance centres was reported to range from 7–14.2%. The designs of these studies varied, however, in terms of sampling period, children’s age classifications and reported medications [14–16, 29]. With the exception of vaccines, the proportion of ADRs related to drugs (8.2%) in this study was slightly higher than that reported worldwide (7.7%) [16]. These ADRs occur in children at all age groups, and are noted to be from the therapeutic class of ‘anti-infectives for systemic use’, particularly vaccines (55.7%), and to cause a wide variety of reactions mainly from the SOC of “application site disorders’ (32.2%). In addition, the time period covered in this study is sufficient to witness the emerging role of nurses in the reporting of ADRs in Malaysia. This is because the nurse is the main healthcare professional that administers vaccine to the patient; especially nurses in primary and secondary healthcare institutes, such as district hospitals and clinics. Meanwhile, the high ADR reporting in the state of Penang as compared to the other states within Malaysia is mainly due to the strong ADR reporting culture among the senior medical specialists and pharmacists in two tertiary public hospitals there. This culture feeds through to junior medical staff, including nurses, who are taught and encouraged to submit ADRs that they encounter.

### Gender and Age Groups

In this study, more than 50% of the ADRs involving children were for females, with an even greater proportion in the oldest age group (12–17 years old). Most of the ADRs (92.7%) reported were related to vaccines. The reason for this dominance was the introduction of Malaysia's cervical cancer prevention programme instituting free HPV immunisation to 13 years old Malaysian girls, which started in 2010 [30]. Other studies have also shown female dominance for ADRs related to vaccine administration [31].

On the contrary, a higher reporting rate for males was reported by Rashed et al. [32]. A similar trend of higher prevalence of ADRs among males, particularly those in the 2 to 11 years age groups, was also observed in two studies that included data from European and Vigibase member countries [16, 31]. Apart from the common prevalence of certain childhood diseases such as asthma and attention deficit hyperactivity disorder among males, no definite explanation for this difference has been identified [16, 31]. Meanwhile, the current study showed a higher prevalence of ADRs among adolescents 12–17 years; in contrast to worldwide Vigibase and Danish data that mostly consist of younger age groups (≤ 2 years old) [16, 17].

### Type of Reporter

The initiatives taken by the NPCB through continuous ADR awareness programmes has resulted in a steady increase of ADR reporting over the years, with the majority of the reports now received from the pharmacists [24, 33]. ADR reporting activity has been included as part of pharmacist training modules, which possibly explains the high reporting rate among pharmacists. Although physicians are traditionally the primary reporters for ADRs in children, this study shows that, in a Malaysian context, nurses reported the most ADRs [16].The involvement of nurses in ADR reporting started in 2009 and this study discovers a dramatic increase in ADRs reporting by the nurses since 2010. Apart from ongoing awareness efforts by the NPCB, the dramatic rise of ADRs in 2010 and 2011 was a result of high reporting of ADRs related to HPV immunisation, as well as the administration of the H1N1 vaccines due to the H1N1 pandemic [34]. The same finding was reported by a study conducted in the UK, where the high volume of reports was attributed to a national immunization programme or campaign [29]. Although overall ADR reports received by the NPCB increased every year, a drop of 40% in ADRs related to vaccine administration was seen in 2012 [35]. Since paediatric ADRs mostly developed following immunisation, the low reporting of ADRs related to vaccines in that particular year has contributed to a drop of ADRs for children in 2012.

Even though, the target of more than 200 reports per million population has been exceeded, the low rate of ADR reporting among private healthcare professionals, in spite its ADRs awareness efforts, is a cause for concern and the NPCB has now extended its ADR programmes to universities and professional medical and nursing associations. Other initiatives include the development of an online reporting system and ADR reporting promotion to community pharmacists through its professional association’s bulletin [24, 35].

A study conducted among physicians in one of the university hospitals in Malaysia revealed that a high proportion of suspected ADRs were not reported due to uncertainty as to the types of reactions to report (81.4%) and a lack of awareness on the importance of ADR reporting (40%) [36]. These findings could now be obsolete, however, since this study was conducted in 2007, and there have been significant efforts to increase awareness among healthcare providers since then. Future study needs to be undertaken to assess the impact of NPCB awareness initiatives, however, and to identify factors that prohibit private healthcare providers from reporting ADR events.

### System Organ Class

Unlike most studies, which utilize the MEdRA for ADR SOC classification, the WHO-ART was used in this study as this classification is used by NPCB in its routine ADRs reporting to WHO-UMC. Nevertheless, similar to other studies, the most frequently reported SOCs in children were related to skin reactions and administration site conditions, nervous system disorders and general disorders [15, 16].

### Anatomical Therapeutic Chemical Classification

The data from the current study revealed that routine administration of vaccines is associated with a large number of ADR reports, dwarfing the number of reports for medications. This reflects the high usage of vaccines in the paediatric population compared with medicines. The high volume of paediatric ADR reports in respect to vaccines has also been noted in other studies reviewing national pharmacovigilance databases [15, 17, 31].

When the data were further broken down to therapeutic group (ATC Level 2), with the exception of vaccines, antibacterial drugs for systemic use appear to be the most prominent drugs suspected of causing ADRs in children, followed by drugs from the nervous system therapeutic group. Indeed, drug utilization studies of children in European countries show that the most commonly prescribed drugs are antibiotics [37]. This finding was also in line with the finding of an exploratory study on paediatric ADRs from the Vigibase system [15–17].

In the current study, the most common drugs associated with ADRs were among the most commonly utilized drugs in Malaysia as reported in the National Medicines Use Survey (NMUS), except for antivirals for systemic use and antineoplastics. The NMUS, which was designed to support the implementation of the National Medicines Policy, collects information on the supply, procurement, prescription, dispensing and use of drugs in Malaysia [38, 39]. Although information from the NMUS can be used for estimating the degree of underreporting of ADRs, and the number of people exposed to certain medications in the occurrence of ADRs, this information is inconsequential for the paediatric population. The NMUS uses Defined Daily Doses (DDD), the assumed average maintenance dose per day, as its main indication in adults in reporting drug utilization, and this makes it impossible to estimate the prevalence of drug use in paediatrics. In addition, the survey only provides information on medicines that have been procured or prescribed or dispensed, and this does not necessarily equate to medication actually consumed by patients. A national survey using information on prescribed daily dosages and indications should be initiated and compared with the DDD values so as to get accurate drug utilization figures for the Malaysian paediatric population [39–41].

### Outcomes from ADRs

Similar to other studies, a large number of the ADRs reported had mild outcomes, with only a small number being severe [18]. The current study revealed that the majority of the fatal ADRs were associated with drug rather than vaccine administration. In Malaysia, there were 32 fatal ADRs (0.28%) during the study period, which was lower than reported in other published literature (0.77% - 1.15%) [15, 17]. Although other studies have shown the majority of fatal cases to occur in younger childhood, this study found more fatalities in adolescents than in younger age groups [15]. The higher proportion of fatal cases in adolescents could be a result of the higher proportion of ADRs reported to the pharmacovigilance centre for this age group which has been shown in an international multicentre study [28]. Even though anticonvulsant administration in a paediatric context has been noted to cause the highest rate of fatalities, there were only four reported fatalities associated with anticonvulsants [42].

This study presents the overall reporting of ADRs for children in the Malaysian ADR database (the QUEST2 system) and only reports the incidence of ADRs in children, types and severity of ADRs and the most common drugs associated with ADRs. The current study presents the first large-scale data on ADR reported in children nationally. Studies monitoring specific childhood age groups, especially in respect to children below 2 years old could provide more useful information since the wider literature has reported more ADRs in this population group than has been shown in the current study.

The variation of risks for ADRs in different childhood age groups, and details regarding the management of the reaction, were not studied however, since some of these data were not readily available. In addition, the possible causal association between a medicine or vaccine and the suspected ADR was not formally assessed since the study only analysed the characteristics of suspected ADRs reported to NPCB. Furthermore, the relationship between the use of off-labelled drugs and the occurrence of ADRs were not studied since the list of off-labelled drugs is not well defined in Malaysia.

The data are derived entirely from the Malaysian ADR spontaneous reporting database and are therefore subject to the limitations of any such system. Those limitations highlighted in the literature include under-reporting of ADRs, variable quality in the completion of the reporting form, reporting biases, inability to calculate the true incidence of any ADRs reported, assessment of causality between a drug and an ADR and difficulty in identifying ADRs with long latency periods following the use of a drug [42–45].

## Conclusions

A sharp increase in ADR reporting in respect to children over the last 14 years was observed in Malaysia. The majority of ADRs reported for children were related to the use of vaccines and anti-infectives in adolescents. In lieu of that, the prevalence of fatality caused by reported ADRs in Malaysia is lower than the benchmark of developed countries. Most suspected ADRs were related to vaccines, which is linked to the emerging role played by nurses in the spontaneous reporting of ADRs.
